# Consumer Survey on the Environmental Impact of Food Packaging and How It Influences Purchasing Decisions

**DOI:** 10.1155/ijfo/9458195

**Published:** 2025-07-01

**Authors:** Cengiz Caner, Melvin A. Pascall, Mehmet Seckin Aday, Dogan Bicki

**Affiliations:** ^1^Department of Food Engineering, Canakkale Onsekiz Mart University, Canakkale, Turkey; ^2^Department of Food Science and Technology, The Ohio State University, Columbus, Ohio, USA; ^3^Department of Public Administration, Faculty of Economics and Administrative Sciences, Muğla Sıtkı Koçman University, Mugla, Turkey

**Keywords:** consumer, decisions, eco-friendly, packaging, purchasing

## Abstract

This study investigated the environmental consciousness of 270 consumers, their behavior towards the purchase of packaged food, their willingness to pay more for products in environmentally friendly containers, and their attitude and knowledge towards recycling and pollution. The data collected were statistically analyzed using Chi-square, *t*-tests, and multinominal and logistic regression analyses. Results showed that consumers preferred glass when compared to other packaging material types. Females were more willing to pay more for products in environmentally friendly packaging. As the respondents' education level increased, the respondents were more knowledgeable about recycling symbols and believed that recycling helped reduce environmental pollution. The income level of the respondents was not conclusive on its effect on the attitude on environmental packaging. Younger respondents were more conscious of recycling symbols and preferred plastic more than the other age groups. Environmental consciousness thus effected the buying decision of the respondents.

## 1. Introduction

Environmentalism has emerged as an important global phenomenon during the last decade owing to an increase in environmentally related concerns and ecological pressures [1, 2]. As a result of these efforts, consumers are becoming more conscious and deliberate about their shopping choices and are increasingly asking for “green” products that provide benefits to their families, lifestyles, and communities. In the past few years, mainstream consumers have shown an interest in environmentally friendly products, and this is a suggestion to manufacturers and retailers that they need to place more of these products in the market. It can be argued that consumers who are increasingly concerned and realize the essentials of environmental issues are considered “green consumers.” They can be described as consumers who make their buying decisions, at least partly on the basis of personal environmental criteria [3, 4].

This increase in environmental concerns shown by consumers has encouraged the provision of more information about the environmental characteristics of a product (a term called “ecolabeling”). Ecolabeling refers to a product's collective overall environmental performance [5]. Ecolabeling has thus become the medium for promoting both the production and consumption of products that are “more environment friendly” when compared with comparable products that are not comparably labeled. Ecolabels are potentially attractive instruments that could serve to inform consumers about the environmental impact of their purchasing decisions, while simultaneously providing producers with a tool for extracting market place preference and thus market share [6, 7].

A knowledge of consumers' awareness and attitude about a product, service, institution, or social issue is essential to the success of a marketing venture. In 2009, [8] reported that, “attitude, as opposed to knowledge and behavior, is the most significant predictor of consumers' willingness to pay more for ecologically favorable products.” Even though consumers may have limited knowledge about environmental sciences they could still have strong emotions for the wellbeing of their immediate living location. This does not mean that knowledge is not important. Indeed, knowledge is an important factor in the decision-making process of consumers. This fact has been emphasized and demonstrated in numerous studies reported in the literature [5, 9, 10].

Our study investigated the environmental conscious behavior of consumers by focusing on the following: (1) their willingness to pay for environmentally friendly packaged products and (2) their willingness to separate garbage from food packaging into material types after consuming the product.

## 2. Material and Methods

### 2.1. Survey Participants

This study used a face-to-face survey that comprised a total of 270 individuals. The participants were 120 males and 150 females (Table 1) in the City of Canakkale, northwestern Turkey. All participants agreed to a 15-min interview and were asked attitudinal questions about their knowledge of how consumers evaluate products and packaging for their environmental friendliness.

The age groups of the participants were 8.1% <20 years old, 52.96% 21–29, 20% 30–39, 14.44% 40–49, 3.33% 50–59, and 1.11% >60 years old. The participants were students (51.85%) and employed adults (26.29%). For the students, 49.25% completed high school and 41.70% had a university degree. Most participants were females (55.55%). The majority (89.25%) lived within the limits of the city. The questions posed to the participants are shown in Table 2.

### 2.2. Survey Measurement

#### 2.2.1. Dependent Variables

The first empirical model was designed to predict consumers' willingness to pay more for environmentally friendly packaged products. The respondents were asked the following question: *“If you decide to purchase an environmentally friendly packaged product, what percent more of the base prices are you willing to pay?”* Responses were recorded and divided into three categories: (1) not willing to pay more, (2) willingness to purchase environmentally friendly products if the price is equal to the regular products, and (3) willingness to pay more. The second model included the dependent variable of the disposal behavior. It measured the responses from the participants to the following question: *“Do you separate food packaging garbage into paper, plastic, glass, and metals and from other household refuge?”* The responses were recorded as dichotomous variables with a yes or a no answer.

#### 2.2.2. Independent Variables

To measure consumers' environmentally consciousness, four items in the dataset were identified. Those items reflecting the individual's level of environmental consciousness, trustfulness, and beliefs about environmentally friendly packaging. The questions were as follows: (1) *“What is the biggest influence on your decision to purchase a new food product?”*, (2) *“Is your decision to purchase a product influenced by the environmental friendliness of the package?”*, (3) *“Do you trust the truthfulness of labels which state that the package is environmentally friendly?”*, and (4) *“Do you believe that package recycling reduces pollution?* The response to each question was recoded as a binary variable. The controlled variables included age, educational attainment, gender, and incomes as categorical variables in each model.

### 2.3. Statistical Analysis

Chi-square analyses and *t*-tests were performed to identify differences in environmental conscious behaviors between groups defined by categorical variables of gender, educational attainment, income level, and age.

In evaluating the first research question, multinomial regression analysis was used to analyze the relationships between a nonmetric dependent variable and dichotomous independent variables. The dependent variable of willingness to pay more for environmentally friendly packaged products was recorded and consisted of three groups: (1) not willing to pay more, (2) willingness to buy environmentally friendly packaged products if they were equal in price to regular products, and (3) willingness to pay more. The group of willingness to buy environmentally friendly products if they were equal in price, as the reference group, was compared to the other two groups.

Logistic regression analysis was used to evaluate the second research question about consumers' behavior towards the disposal of food packaging after consuming the product. With the dichotomous responses of 1 (*yes*) and 0 (*no*) as a predictor variable, the results of logistic regression analyses provided the probability score of engaging in disposal behavior and not engaging in it, respectively. These statistical analyses were conducted with SPSS Version 20 software program.

## 3. Results and Discussion

### 3.1. General Responses to Survey Questions

For Question #1, 88.14% of the responses felt that purchasing food in a package was important. A total of 11.86% of the respondents to this question felt that it was not important to them if the food was packaged. These results corroborated with the results from Question #2 where 52.96% of the respondents said that they preferred to shop at large grocery stores instead of the farmers' market (14.81%). Most foods in large grocery stores are marketed as packaged products, and the package is used as a marketing tool. In the case of farmers' markets, most foods are not packaged but are sold as fresh produce such as fruits, root crops, and vegetables. In the farmers' markets, packaging as a marketing tool is less emphasized.

Question #3 provided more information about the influence of packaging on the decision to purchase a product. The results showed that 24.07% of the respondents will purchase a specific product if the packaged commodity could be recognized from prior advertising. A total of 11.11% of the respondents acknowledged that they were influenced by the attractiveness of the packaging. However, most of the respondents said that the package label guided them to products having natural as compared to artificial food additives. This issue of food additives seemed to be closely associated with the safety of the packaged product because 27.03% of the respondents said that they would respond positively to products with labels that provided assurances about the product's safety. Since only 5.9% of the respondents indicated that they would be influenced by the environmental friendliness of the packaging, it was concluded that the majority will not put this before the safety of the food product.

When the question of environmental friendliness of a package was asked (Question #4) and the issue was separated from the safety of the product, the results showed that 55.18% will choose a package that is more environmentally friendly than one that is not (6.66%). However, 32.22% indicated that they will put the price of the product above its environmental friendliness when deciding to purchase it.

This response led to Question #5, “when shopping for food, how much more are you willing to pay for a product in a container that is environmentally friendly, compared with one that is not?” In response, 56.66% of the respondents indicated that if the prices of two assorted brands of a given food product were identical, they will choose the one packaged in a container that was more environmentally friendly. These individuals were not categorized as wanting to pay, or not wanting to pay more for an environmentally friendly product. However, 16.29% said that they will not pay more for a product based on its environmental friendliness. The results showed that 22.59% said that they would be happy to pay as much as 10% more, but only 3.33% and 1.11% would pay 20% and 30% more, respectively, for an environmentally friendly product. From these results, it was seen that 72.95% of the respondents (that is, 56.66% + 16.29%) did not indicate that they were willing to pay more for an environmentally friendly product.

Question #6 sought to determine the perceptions of the participants towards distinct types of packaging materials. The results showed that 86.29% preferred to purchase food in glass containers, compared with only 5.92% who chose plastic, 1.11% who favored fabric, and <1% indicating metal. A total of 6.29% said that they had no preference for the packaging material type. This result indicated that consumers perceived glass to be a better packaging material and more environmentally friendly when compared with other materials. This was so because 55.18% (a majority) of the respondents to Question #4 indicated that they preferred environmentally friendly packaging. To reinforce this point, the data (to be discussed later) also showed that irrespective of the education level, all respondents selected glass as their packaging material of choice and metal as the one least liked.

Question #7 focused on package recycling and recycling symbols. The majority (82.22%) of the respondents indicated that they were familiar with these symbols. Question #8 inquired if the participants believed that the information printed on a package was truthful when it claimed that the package was environmentally friendly. In response to this, 21.48% indicated a strong yes and 52.96% said yes. This meant that 74.44% of the respondents trusted the environmental information on the labels. For the remainder of the responses, only 3.7% did not trust the information and 21.85% were uncertain if such information was authentic.

Question #9 inquired about the number of participants that were actively engaged in recycling. The data collected showed that 53.3% were not actively participating in sorting waste packaging according to individual material types. Only 11.11% indicated that they always sorted their waste packaging, and 35.55% sorted occasionally.

Question #10 inquired if the participants believed that the effort to recycle waste packaging was truly effective in reducing environmental pollution. The responses showed that 44.07% strongly believed that recycling was effective and 51.85% indicated that recycling had a positive effect on pollution reduction. Thus, 95.92% of the respondents had a positive view of recycling and that it had the potential to reduce pollution.

In the last question (Question #11) shown in Table 2, the focus was to determine the types of commodities and the priority in which the cohorts purchased them. The results showed that the participants chose food as the most purchased commodity when all the participants were considered as a distinct group. The data showed that 55.50% of all incomes were spent on food. For clothing, education, and miscellaneous items, there seemed to be a similar distribution of the income. This made this study more urgent and relevant.

### 3.2. Influence of Gender on Purchases

Table 3 shows the influence of gender on the decision to purchase a product. Females (92.55%) were more influenced by the package of the product when deciding to purchase it, when compared with males (83.49%). Also, 16.51% of the males said that the package was not important when compared with 7.45% of the females. These results also corroborated with the results for the influence of advertising on the purchasing decisions. The data showed that females (27.33%) were more influenced by advertising, when compared with 22.94% of the males. Females (13.66%) were also influenced more by the attractiveness of the package when compared with men at 11.01%.

On the influence of gender, the data showed that 26.85% of the male respondents will not pay more for a product even though they knew that it was packaged in an environmentally friendly container. However, only 9.32% of the females responded similarly. Thus, females were more willing to pay more for a product packaged in an environmentally friendly container. The data also showed that 59.01% of the females, compared with 50.93% of the males, will choose an environmentally friendly package when presented with two packages having the same price, and one was not environmentally friendly. At the same time, 26.71% of the females were willing to pay 10% more for a product within an environmentally friendly package, when compared with only 16.67% of males. There were minor differences between the responses for the willingness to pay 20% and 30% more for an environmental package. Few respondents were willing to pay that much for such products. Data from this question corroborated with those from Question #4, which asked if an individual's decision to purchase a product was influenced by the environmental friendliness of the package. The data showed that females (61.49%) preferred to purchase products in environmentally friendly packages, when compared with the males at 50.48%.

On the question of package recycling, both men and women seemed to similarly (*p* > 0.05) perform the separation of trash based on the packaging material types, but more women were familiar with the recycling symbols than men. Both males and females also equally seemed to believe that the recycling of packaging materials helped reduce environmental pollution. For all other responses, there was no significant (*p* > 0.05) difference in the data between the sexes.

Many studies have shown significant differences between men and women in environmental attitudes [11, 12], with men having more negative attitudes towards the environment when compared with women [13]. Women were more likely to buy green products because they believed that such products were better for the environment [14]. This finding is similar to the results of our study, which showed significant differences in the responses between males and females to the questions on the importance of packaging, their preference for environmentally friendly packaging and their awareness of the recycling symbols. The logistic regression analyses showed that gender did not significantly explain the variance in one's willingness to pay for an environmentally friendly packaged product. The multinomial regression analyses in Table 4 showed that gender played a significant role in differentiating the willingness to pay more from the willingness to pay, if the price was equal (reference group). Compared to females, the data showed that males were 73.7% less likely to pay more for an environmentally friendly package (*e*^*β*^ = 0.263, *p* < 0.01).

### 3.3. Influence of Educational Attainment on Purchases

When the education level of the respondents was considered, Table 5 showed that 65% of those having a graduate level university degree preferred to do their shopping at large grocery stores. None in this category indicated a preference to shop in farmers' markets, and 35% said that they preferred to shop at other types of markets. For the participants with an undergraduate university degree, 67.1% selected large grocery stores, 10% indicated their preference for farmers' markets, and 22.9% selected other types of markets. For university students, the data showed that 56.1% chose large groceries, 15.9% chose the farmers' markets, and 28% other market types. This trend in obtaining a more evenly spread in the choices between the markets increased as the level of education fell, except for the primary level educated individuals. For the high school level educated individuals, 50% selected large grocery stores, while 33.3% chose the farmers' markets and 16.7% said the other types of markets. For the junior high school graduates, 42.9% chose the large groceries but 28.6% selected both the farmers' markets and the other types of markets. In the case of the primary level educated individuals, 100% selected the farmers' markets. In summary, the data based on education levels of the participants indicated that, as the level of education increased, the individuals were more inclined to do their grocery shopping at large grocery stores and less so at farmers' markets.

When asked about their preference in purchasing a product that was packaged when compared with the same product sold as an unpackaged unit, none of the university graduates said that they would choose the unpackaged product. For the high school graduates, the survey showed that they did not have as strong an opinion on the packaging of a product. The data for the high school graduates showed that 49.57% preferred food packaged in glass containers and 51.61% were willing to pay >10% than the base price of a product if it were packaged in an environmentally friendly container.

The data also showed that the choice of packaging material type was influenced by the education status of the recipients. A total of 90.24% of the respondents with master's degrees indicated that they preferred to purchase food in glass containers when compared with materials of the other types. This was an overwhelming choice by this category since only 7.32% of the master's graduates selected plastic, 2.44% said that they had no clear preference, and none indicated that they would choose fabric or metal over the other types of materials. Of the recipients with bachelor's degrees, 88.73% chose glass packaging, 4.23% indicated plastic, 1.41% selected fabric, 5.63% said that it did not matter, and none chose metal packaging as their first choice. When the data from the high school graduates were considered, the results differed from those of the master's and bachelor's degree graduates. For the high school graduates, the pattern of choosing was similar to those who had university degrees. The data showed that 61.54% chose glass, 15.38% selected both plastic and fabric, 7.7% had no preference, and none indicated that they would choose metal containers.

The results of this study showed that, as the level of education attained by the respondents increased, they appeared more willing to pay more for a product packaged in an environmentally friendly container. As the education levels increased from primary to postgraduate, there was more willingness to pay more for such packaged products. The only exception was the data from the junior high school respondents, where 16.67% said that they would pay up to 30% more for a product in an environmentally friendly package. There was suspicion that this may have been recorded in error.

The data also showed that, as the education level increased from primary to postgraduate, the respondents were more aware of the presence and meaning of the recycling symbols on packages. This also explained why a similar trend was observed for the practice of separating trash according to the packaging types. As the education level of the respondents increased, the practice of separating trash, trusting the truthfulness of labels, and the belief that recycling had a positive influence on reducing pollution also appeared to significantly (*p* < 0.05) increase.

The results obtained in our study corroborated with those of Schwartz and Miller [15] and Newell and Green [16], where the relationship between education and the behavior of green consumers was positively correlated. Low education (elementary, junior high, and high school) levels had lower correlated responses to environmentally favorable products. As a result, it was assumed that highly educated people would be more inclined to positively relate to environmental issues. Based on the results of the logistic and multinomial regression analyses, educational attainment was a significant predictor of both types of environmentally conscious behaviors in our study.

### 3.4. Influence of Income on Purchases

For the influence of income on purchasing, the data showed that increasing income levels, from 0.5 to 1.0 k to a high of >2.5 k per year, positively correlated with willingness to purchase food that were packaged instead of unpackaged items. The data showed that 81.40% of the 0.5–1.0 k group said that they will purchase food in packages while 82.86%, 95.35%, 96.55%, and 90.79% of the 1.0–1.5 k, 1.6–2.0 k, 2.0–2.5 k, and >2.5 k groups, respectively, will choose packaged over unpackaged food. The same trend was also seen for the choice of market types. The data also showed that the higher the income level, the less inclined the participants were to shop at the farmers' markets. For example, for those in the income bracket 0.5 to 1 k, 24.42% chose the farmers' markets while 2.33%, 6.89%, and 14.47% in income levels <2, <2.5, and >2.5 k, respectively, chose the farmers' markets.

When presented with Question #3 which asked, “what has the biggest influence on your decision to purchase a new food product,” the response patterns from all income levels were similar. Except for income level 2.0–2.5 k, the other groups were similarly influenced by advertisement. This 2–2.5 k income group was influenced more by advertising. The highest (>2.5 k) and the lowest (0.5–1 k) income groups were more positively influenced by the attractiveness of packaging when compared with the other groups. All income groups, except the 1.6–2.0 k, relied on the label information more than their knowledge of the safety of a product when deciding if to purchase it. For the 1.6–2.0 k group, 41.86% chose their prior knowledge of food safety but 23.26% selected the label information. This was the same group that was least influenced by the attractiveness of the packaging when deciding if to purchase a product. For all income groups, the combined selection of label information and prior knowledge of food safety were significantly higher than the other choices combined.

All income groups chose glass as the most liked packaging material and metal as the least. The distribution of the percentages for their willingness to pay more for environmentally friendly packaging was similar for all income groups. The lowest and highest income earners were less familiar with the recycling symbols when compared with the middle-income groups (Table 6). There were no significant differences (*p* > 0.05) in the responses from the lowest and highest and the 2.0–2.5 k income groups when they were asked about their involvement in separating garbage according to packaging material types. These groups showed less inclination to separate their garbage when compared with the 1.0–1.5 and the 1.6–2.0 k groups. When asked if they trusted the truthfulness of the environmental information printed on package labels, the highest income group (>2.5 k) was the least trusting. This result was also reflected in the response of this group to the question of recycling and its contribution to pollution. This highest income group was least convinced that recycling helped to reduce pollution.

Reports in the literature about the relationship between income levels and environmental concerns seemed to vary in consistency. In 1996, Roberts [17] found no significant relationship between income and environmental concerns. However, Qiao and Dowell [18] indicated that there were significant relationships between income and environmental attitudes and behavior. Also, in 2017, Yoo et al. [19] reported that income levels could influence the decision-making process. Our study showed that consumer attitudes towards environmental awareness were generally positive; however, we were inclined to conclude that it was not always consistent. The logistic and multinomial regression analyses did not confirm the significance of income in neither the willingness to pay more for environmentally friendly packaged food nor engaging in separating food packaging garbage into paper, plastic, glass, and metals (Table 7).

### 3.5. Influence of Age on Purchases

Data on the influence of age and purchasing behavior are shown in Table 8. Older individuals (>50 years old) were more inclined to purchase packaged products when compared to the same product, but unpackaged. However, this same age group preferred to shop for food at farmers' markets more that their younger counterparts. The younger age groups preferred to shop at grocery stores and other avenues of vending. Individuals <40 years old were influenced more by advertising when compared with older individuals. Except for the age group >60 years old, younger people (<30 years) were influenced more by the attractiveness of a package when deciding to purchase a product. For the <20 and 20–29 age groups, 14.29% and 15.54%, respectively, were influenced by the attractiveness of the package, when compared to 3.85% and 5.41% of the 30–39- and 40–49-year-olds, respectively. Younger people were less trusting and showed less interest in reading package labels when compared to older folks. The younger individuals were more conscious of food safety issues but less concerned about the environmental friendliness of the package. The only age group that showed significant interest (*p* < 0.05) in the environmental friendliness of the package was the >60-year-old individuals. All age groups chose glass packaging more than the other types of materials. However, younger people, especially those <20 years old, were more inclined to choose plastics. A majority in each of the age groups indicated that they would choose the environmentally positive packaging if they had a choice between two products having the same price but one was environmentally packaged and the other was not. However, older individuals were less inclined to pay more than 20% for an environmentally packaged product when compared with younger people (<40 years old). Younger individuals were more conscious of recycling symbols on food packages and seemed more inclined to separate their garbage into materials of distinct types when compared with older age groups. Although some of the individuals <20 years old had some uncertainty, all age groups believed that recycling helped reduce pollution. The older individuals spent more on food and clothes than younger people, but older individuals spent significantly (*p* < 0.05) less on education.

## 4. Conclusions

This study showed that consumers preferred glass packaging more than other types of materials. Gender influenced the willingness to pay more for products in environmental packaging. As the education level of consumers increased, they knew more about the recycling symbols and believed that recycling helped reduce environmental problems. Income and education levels had some influence on purchases, but they were not conclusive for all categories of income and educational attainments. Younger people preferred plastic more than the other age groups and they were more conscious of the recycling symbols. Younger people and females were influenced more by the attractiveness of the package.

## Figures and Tables

**Table 1:** Demographic profile of respondents.

	**Frequency**	**Percentage**
Gender		
Female	150	55.50
Male	120	44.50
Age		
20 and under	22	8.10
20–29	143	52.96
30–39	54	20
40–49	39	14.44
50–59	9	3.33
60 and above	3	1.11
Marital status		
Married	86	31.85
Divorce	8	2.96
Single	176	65.18
Number of children		
None	198	73.33
One	34	12.59
Two	32	11.85
Three	5	1.85
Four and above	1	0.37
Level of education		
Less than elementary	6	2.22
Elementary	7	2.59
Middle school	11	4.07
High school	133	49.25
Bachelor's degree	65	24
Master's degree	48	17.70
Occupation		
Farmer	2	0.70
Civil servant	71	26.29
Factory worker	10	3.70
Retired	5	1.85
Tradesmen	1	0.37
Student	140	51.85
Others	41	15.18
Monthly income (Turkish Lira)		
500–999	90	33.30
1000–1499	35	12.96
1500–1999	43	15.92
2000–2500	27	10
>2500	75	27.77
Area of residence		
City	241	89.25
County	24	8.88
Village–rural	3	1.11

**Table 2:** Survey questions and participants' responses.

**Question number**	**Question prompt**	**Responses**	
		**Frequency**	**Percentage**
1. Is your decision to purchase a food product influenced if it is packaged or not packaged?	1. Packaging not important	32	11.86
	2. Packaging important	238	88.14
2. Where do you mainly do your food shopping?	1. Farm markets	40	14.81
	2. Big markets	155	52.96
	3. Others	75	20.00
3. What has the biggest influence on your decision to purchase a new food product?	1. Advertisement	65	24.07
	2. Attractive packaging	30	11.11
	3. Label information (natural ingredients)	86	31.85
	4. Food safety	73	27.03
	5. Environmentally friendly	16	5.90
4. Is your decision to purchase a product influenced by the environmental friendliness of the package?	1. No, is not important	18	6.66
	2. No, price is important	87	32.22
	3. Yes, I prefer environmentally friendly packages	149	55.18
	4. Others	16	5.92
5. When you have different alternatives for food packaging, what kind of packaging materials do have preferred?	1. Glass	233	86.29
	2. Plastic	16	5.92
	3. Fabric	3	1.11
	4. Metal	1	0.37
	5. It does not matter	17	6.29
6. If you decide to purchase an environmentally friendly packaged product, what percent more of the base price are you willing to pay?	1. Will not pay more. I prefer the lower cost packaged prod't	44	16.29
	2. If 2 prod'ts equal in price, will choose environment friendly prod't	153	56.66
	3. Willing to pay >10% for environment friendly package/prod't	61	22.59
	4. Willing to pay >20% for environment friendly package/prod't	9	3.33
	5. I am willing to pay 30% more for environmentally friendly packaged product	3	1.11
7. Are you familiar with the following symbols? 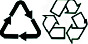	1. Yes	222	82.22
	2. No	48	17.78
8. Do you separate food packaging garbage into paper, plastic, glass, and metals and from other household garbage?	1. Yes, all the time	30	11.11
	2. Sometimes	96	35.55
	3. No	144	53.30
9. Do you trust the truthfulness of labels which state that the package is environmentally friendly?	1. Certainly yes	58	21.48
	2. Yes	143	52.96
	3. Certainly not	10	3.70
	4. I am not sure	59	21.85
10. Do you believe that package recycling reduces pollution?	1. Certainly yes	119	44.07
	2. It makes a positive input	140	51.85
	3. Certainly not	5	1.85
	4. I do not know	6	2.22
11. What part of your income is spent on the following?	1. Food	150	55.50
	2. Clothing	42	15.50
	3. Education	33	12.22
	4. Others	45	16.66

**Table 3:** Influence of gender on purchasing decisions.

**Influence of sexual orientation by percentage**	**Males**	**Females**	**t** ** value**
Question # 1—packaging			
1. Packaging not important	16.51	7.45	
2. Packaging important	83.49	92.55	**t** = −1.62^∗∗^

Question # 2—market types			
1. Farmers' market	12.04	11.18	
2. Large grocery store	62.96	62.11	
3. Others	25.00	26.71	*t* = 0

Question #3—influence			
1. Advertisement	22.94	27.33	
2. Attractive packaging	11.01	13.66	
3. Label information	31.19	31.06	
4. Food safety	32.11	27.33	
5. Environmentally friendly	2.75	0.62	*t* = 1.56

Question #4—environment friendly			
1. No, is not important	7.62	7.45	
2. No price is important	40.95	29.19	
3. Yes, I prefer environ. friendly packaging	50.48	61.49	
4. Others	0.95	1.86	

Question #5—packaging materials			
1. Glass	80.73	90.68	
2. Plastic	6.42	5.59	
3. Fabric	0.92	1.24	
4. Metal	0.92	0.00	
5. Others	11.01	2.48	**t** = 2.76^∗∗∗^

Question #6—will paying more			
1. Will not pay more. Prefer low price pkg.	26.85	9.32	
2. Will choose environ pkg. if lower price	50.93	59.01	
3. Will to pay 10% more for environ pkg.	16.67	26.71	
4. Will to pay 20% more for environ pkg.	2.78	4.35	
5. Will to pay 30% more for environ pkg.	2.78	0.62	*t* = −2.33

Question #7—recycling symbol			
1. Yes	77.06	85.71	
2. No	22.94	14.29	**t** = −1.23^∗^

Question #8—separate garbage			
1. Yes, all the time	13.76	11.18	
2. Sometimes	49.54	49.69	
3. No	36.70	39.13	*t* = −1.33

Question #9—trust label information			
1. Certainly yes	22.94	19.88	
2. Yes	58.72	52.80	
3. Certainly no	0.00	3.73	
4. I am not sure	18.35	23.60	*t* = −1.12

Question #10—recycling and pollution			
1. Certainly yes	49.54	48.45	
2. It makes a positive input	48.62	46.58	
3. Certainly not	0.92	1.86	
4. I do not know	0.92	3.11	**t** = 0^∗∗∗^

*Note:* The star associated with each bold entry corresponds with the statistical significance at the bottom of the table.

⁣^∗^*p* < 0.05, ⁣^∗∗^*p* < 0.01, and ⁣^∗∗∗^*p* < 0.001 indicate statistical significance.

**Table 4:** Multinomial logistic regression: parameter estimates and odds ratios for willingness to pay and not to pay more for environmentally friendly packaged products.

**Predictor**	**Not willing to pay more**			**Willingness to pay more**		
	**β** ** (SE)**	**e** ^**β**^ ** (odds ratio)**	**Wald**	**β** ** (SE)**	**e** ^**β**^ ** (odds ratio)**	**Wald**
Age						
10–19	−1.463 (0.916)	0.232	2.549	−0.824 (0.721)	0.439	1.304
20–29	−0.16 (0.591)	0.984	0.001	0.617 (0.476)	1.854	1.681
40–49	1.113 (0.731)	3.044	2.321	0.963 (0.584)	2.620	2.720
50–59	−0.618 (1.046)	0.539	0.349	15.66 (1183)	6,023,265.6	0.000
60–69	15.287 (0.000)	4,354,664	.	−14.293	6.204e − 007	0.000
Education						
Primary	−**0.073 (1.413)**⁣^∗^	0.929	0.003	14.279 (1094.48)	1,589,475.9	0.000
Jr. high school	0.850 (1.451)	2.339	0.343	−14.251 (1025.02)	6.472e − 007	0.000
Undergraduate	1.558 (0.844)	4.751	3.405	−14.893 (1025.02)	3.405e − 007	0.000
Bachelor	1.601(0.933)	4.959	2.947	−15.211 (1025.02)	2.478e − 007	0.000
Postgraduate	0.288 (1.084)	1.333	0.070	−15.575 (1025.02)	1.721e − 007	0.000
Gender						
Male	−**1.336 (0.451)**⁣^∗∗^	0.263	8.758	0.468 (0.356)	1.597	1.731
Income						
0.5–1 k	−0.353 (0.840)	0.702	0.177	0.329 (0.546)	1.390	0.364
1–1.5 k	−1.368 (0.837)	0.255	2.674	0.050 (0.593)	1.051	0.007
2–2.5 k	−0.656 (0.846)	0.519	0.601	1.088 (0.648)	2.969	2.820
2.5 k and above	−0.043 (0.857)	0.958	0.003	0.628 (0.538)	1.874	1.362
Env conscious 1^1)^	15.307 (1749.48)	4,444,083.4	0.000	−1.099 (1.351)	0.333	0.661
Env conscious 2^2)^	**1.493 (0.459)**⁣^∗∗^	4.450	10.581	−**0.831 (0.363)**⁣^∗∗^	0.436	5.229
Env conscious 3^3)^	0.837 (0.450)	2.311	3.461	−0.722 (0.425)	0.486	2.895
Env conscious 4^4)^	−0.809 (0.931)	0.445	0.754	−1.231 (1.241)	0.292	0.984
Intercept	−32.347 (1749.49)		0.000	27.790 (3907.64)		0.000
*R* ^2^ (Nagelkerke)	0.377					
*R* ^2^ (Cox & Snell)	0.323					
−2LL	284.810					
*χ* ^2^	103.542⁣^∗∗∗^					
*df*	38					

*Note*: Reference category is to choose an environment friendly product if two products are equal in price. The star associated with each bold entry corresponds with the statistical significance at the bottom of the table.

⁣^∗^*p* < 0.05, ⁣^∗∗^*p* < 0.01, and ⁣^∗∗∗^*p* < 0.001 indicate statistical significance.

**Table 5:** Influence of education levels on purchasing decisions.

	**Education levels**						
	**Primary school**	**Jr. high school**	**High school**	**Under graduate student**	**Bachelor degree**	**Post graduate**	**χ** ^2^
Question # 1—packaging							
1. Packaging not important	6 (100)	1 (14.3)	3 (25)	13 (9.8)	9 (12.7)	0 (0)	
2. Packaging important	0 (0)	6 (85.7)	9 (75)	120 (90.2)	62 (87.3)	41 (100)	
	6 (22)	7 (2.6)	12 (4.4)	133 (49.3)	71 (26.3)	41 (15.2)	**52.76**⁣^∗∗∗^

Question # 2—market types							
1. Farmers' market	6 (100)	2 (28.2)	4 (33.3)	21 (15.8)	7 (9.9)	0 (0)	
2. Large grocery store	0 (0)	3 (42.9)	6 (50.0)	72 (54.1)	47 (66.2)	27 (65.9)	
3. Others	0 (0)	2 (28.6)	2 (16.7)	40 (30.1)	17 (23.9)	14 (34.1)	
	6 (2.2)	7 (2.6)	12 (4.4)	133 (49.3)	71 (26.3)	41 (15.2)	**49.36**⁣^∗∗∗^

Question #3—influence							
1. Advertisement	3 (50)	3 (42.9)	3 (25)	34 (25.6)	15 (21.4)	10 (24.4)	
2. Attractive packaging	1 (16.7)	0 (0)	2 (16.7)	21 (15.8)	6 (8.6)	1 (2.4)	
3. Label information	1 (16.7)	2 (28.6)	7 (58.3)	44 (33.1)	24 (34.3)	14 (34.1)	
4. Food safety	0 (0)	1 (14.3)	0 (0)	32 (24.1)	25 (35.7)	15 (36.6)	
5. Environmentally friendly	1 (16.7)	1 (14.3)	0 (0)	2 (1.5)	0 (0)	1 (2.4)	
	6 (2.2)	7 (2.6)	12 (4.5)	133 (49.3)	70 (26.0)	4 (15.2)	**35.82**⁣^∗^

Question #4—environment friendly							
1. No, is not important	2 (33.3)	3 (42.9)	2 (16.7)	2 (1.5)	9 (12.7)	1 (2.4)	
2. No price is important	4 (66.7)	2 (28.6)	4 (33.3)	51 (38.6)	19 (26.8)	10 (24.4)	
3. Yes, I prefer environ. friendly packaging	0 (0)	1 (14.3)	4 (33.3)	76 (57.6)	42 (59.2)	30 (73.2)	
4. Others	0 (0)	1 (14.3)	2 (16.7)	3 (2.3)	1 (1.4)	0 (0)	
	6 (2.2)	7 (2.6)	12 (4.4)	133 (49.3)	71 (26.3)	41 (15.2)	**73.38**⁣^∗∗∗^

Question #5—packaging materials							
1. Glass	6 (100)	4 (57.1)	7 (58.3)	115 (86.5)	63 (88.7)	37 (90.2)	
2. Plastic	0 (0)	0 (0)	3 (25)	8 (6)	3 (4.2)	3 (7.3)	
3. Fabric	0 (0)	1 (14.3)	1 (8.3)	0 (0)	1 (1.4)	0 (0)	
4. Metal	0 (0)	0 (0)	0 (0)	1 (100)	0 (0)	0 (0)	
5. Others	0 (0)	2 (28.6)	1 (8.3)	9 (6.8)	4 (5.6)	1 (2.4)	
	6 (2.2)	7 (2.6)	12 (4.4)	133 (49.3)	71 (26.3)	41 (15.2)	**36.78**⁣^∗∗^

Question #6—will paying more							
1. No. Prefer low price pkg.	2 (33.3)	1 (16.7)	6 (50.0)	18 (13.6)	9 (12.7)	8 (19.5)	
2. Buy env'n pkg. if lower price	4 (66.7)	4 (66.7)	6 (50.0)	77 (58.3)	41 (57.7)	17 (41.5)	
3. Pay 10% more for env'n pkg.	0 (0)	0 (0)	0 (0)	32 (24.2)	19 (26.8)	10 (24.4)	
4. Pay 20% more for env'n pkg.	0 (0)	0 (0)	0 (0)	3 (2.3)	1 (1.4)	6 (14.6)	
5. Pay 30% more for env'n pkg.	0 (0)	1 (16.7)	0 (0)	1 (0.8)	1 (1.4)	0 (0)	
	6 (2.2)	6 (2.2)	12 (4.5)	133 (49.3)	71 (26.5)	41 (15.3)	**49.53**⁣^∗∗^

Question #7—recycling symbol							
1. Yes	0 (0)	1 (14.3)	8 (66.7)	117 (88.0)	60 (84.5)	36 (87.8)	
2. No	6 (100.0)	6 (87.5)	4 (33.3)	16 (12.0)	11 (15.5)	5 (12.5)	
	6 (2.2)	7 (2.6)	12 (4.4)	133 (49.3)	71 (26.3)	41 (15.2)	**55.98**⁣^∗∗∗^

Question #8—separate garbage							
1. Yes, all the time	0 (0)	1 (14.3)	2 (16.7)	16 (12.0)	8 (11.3)	5 (12.2)	
2. Sometimes	1 (16.7)	1 (14.3)	5 (41.7)	70 (52.6)	37 (52.1)	22 (53.7)	
3. No	5 (83.3)	5 (71.4)	5 (41.7)	47 (35.3)	26 (36.6)	14 (34.1)	
	6 (2.2)	7 (2.6)	12 (4.4)	133 (49.3)	71 (26.3)	41 (15.2)	10.44

Question #9—trust label information							
1. Certainly yes	0 (0)	1 (14.3)	5 (41.7)	30 (22.6)	11 (15.5)	9 (22.0)	
2. Yes	6 (100)	4 (57.1)	4 (33.3)	77 (57.9)	40 (56.3)	18 (43.9)	
3. Certainly no	0 (0)	0 (0)	0 (0)	0 (0)	5 (7.0)	1 (2.4)	
4. I am not sure	0 (0)	2 (28.6)	3 (25.0)	26 (19.5)	15 (21.1)	13 (31.7)	
	6 (2.2)	7 (2.6)	12 (4.4)	133 (49.3)	71 (26.3)	41 (15.2)	24.08

Question #10—recycling & pollution							
1. Certainly yes	2 (33.3)	3 (42.9)	6 (50.0)	65 (48.9)	36 (50.7)	19 (46.3)	
2. It makes a positive input	4 (66.7)	3 (42.9)	4 (33.3)	66 (49.6)	31 (43.7)	21 (51.2)	
3. Certainly not	0 (0)	0 (0)	0 (0)	1 (0.8)	2 (2.8)	1 (2.4)	
4. I do not know	0 (0)	1 (14.3)	2 (16.7)	1 (0.8)	2 (2.8)	0 (0)	
	6 (2.2)	7 (2.6)	12 (4.4)	133 (49.3)	71 (26.3)	41 (15.2)	22.17

*Note*: frequency (%), *N* = 270. The star associated with each bold entry corresponds with the statistical significance at the bottom of the table.

⁣^∗^*p* < 0.05, ⁣^∗∗^*p* < 0.01, and ⁣^∗∗∗^*p* < 0.001 indicate statistical significance.

**Table 6:** Influence of income level on purchasing decisions.

	**Influence of income by percentage**					
	**0.5–1 k**	**1–1.5 k**	**1.6–2 k**	**2–2.5 k**	**>2.5 k**	**χ** ^2^
Question # 1—packaging						
1. Packaging not important	16 (18.6)	6 (17.1)	2 (4.7)	1 (3.4)	7 (9.2)	
2. Packaging important	70 (81.1)	29 (82.9)	41 (95.3)	28 (96.6)	69 (90.8)	
	86 (32.0)	35 (13.0)	43 (16.0)	29 (10.8)	76 (28.3)	9.26

Question # 2—market types						
1. Farmers' market	21 (24.4)	5 (14.3)	1 (2.3)	2 (6.9)	11 (14.5)	
2. Large grocery store	39 (45.3)	25 (71.4)	33 (76.7)	19 (65.5)	39 (51.3)	
3. Others	26 (30.2)	5 (14.3)	9 (20.9)	8 (27.6)	26 (34.2)	
	86 (32.0)	35 (13.0)	43 (16.0)	29 (10.8)	76 (28.3)	**22.43**⁣^∗∗^

Question #3—influence						
1. Advertisement	22 (25.6)	7 (20.6)	12 (27.9)	11 (37.9)	16 (21.1)	
2. Attractive packaging	12 (14.0)	3 (8.8)	1 (2.3)	2 (6.9)	13 (17.1)	
3. Label information	31 (36.0)	17 (50.0)	10 (23.3)	9 (31.0)	25 (32.9)	
4. Food safety	19 (22.1)	6 (17.6)	18 (41.9)	7 (24.1)	22 (28.9)	
5. Environmentally friendly	2 (2.3)	1 (2.9)	2 (4.7)	0 (0)	0 (0)	
	86 (32.0)	35 (13.0)	43 (16.0)	29 (10.8)	76 (28.3)	23.00

Question #4—environment friendly						
1. No, is not important	8 (9.3)	1 (2.9)	4 (9.3)	2 (6.9)	4 (5.3)	
2. No price is important	34 (39.5)	15 (42.9)	12 (27.9)	7 (24.1)	22 (28.9)	
3. Yes, I prefer environ. friendly packaging	40 (46.5)	19 (54.3)	27 (62.8)	19 (65.5)	48 (63.2)	
4. Others	4 (4.7)	0 (0)	0 (0)	0 (0)	2 (2.6)	
	86 (32.0)	35 (13.0)	43 (16.0)	29 (10.8)	76 (28.3)	21.44

Question #5—packaging materials						
1. Glass	73 (84.9)	25 (71.4)	41 (95.3)	25 (86.2)	67 (88.2)	
2. Plastic	6 (7.0)	4 (11.4)	1 (2.3)	2 (6.9)	4 (5.3)	
3. Fabric	1 (1.2)	0 (0)	0 (0)	1 (3.4)	1 (1.3)	
4. Metal	0 (0)	1 (2.9)	0 (0)	0 (0)	0 (0)	
5. Others	6 (7.0)	5 (14.3)	1 (2.3)	1 (3.4)	4 (5.3)	
	86 (32.0)	35 (13.0)	43 (16.0)	29 (10.8)	76 (28.3)	18.27

Question #6—will paying more						
1. Will not pay more. Prefer low price pkg.	15 (17.4)	8 (22.9)	4 (9.5)	8 (27.6)	9 (11.8)	
2. Will choose environ pkg. if lower price	51 (59.3)	18 (51.4)	21 (50.0)	15 (51.7)	44 (57.9)	
3. Will to pay 10% more for environ pkg.	18 (20.9)	8 (22.9)	12 (28.6)	3 (10.3)	20 (26.3)	
4. Will to pay 20% more for environ pkg.	2 (2.3)	0 (0)	4 (9.5)	2 (6.9)	2 (2.6)	
5. Will to pay 30% more for environ pkg.	0 (0)	1 (2.9)	1 (2.4)	1 (3.4)	0 (0)	
	86 (32.0)	35 (13.0)	43 (16.0)	29 (10.8)	76 (28.3)	23.01

Question #7—recycling symbol						
1. Yes	66 (76.7)	27 (74.3)	36 (83.7)	26 (89.7)	67 (88.2)	
2. No	20 (23.3)	9 (25.7)	7 (16.3)	3 (10.3)	9 (11.8)	
	86 (32.0)	35 (13.0)	43 (16.0)	29 (10.8)	76 (28.3)	6.25

Question #8—separate garbage						
1. Yes, all the time	4 (4.7)	6 (17.1)	7 (16.3)	7 (24.1)	8 (10.5)	
2. Sometimes	43 (50.0)	18 (51.4)	22 (51.2)	11 (37.9)	42 (55.3)	
3. No	49 (45.3)	11 (31.4)	14 (32.6)	11 (37.9)	26 (34.2)	
	86 (32.0)	35 (13.0)	43 (16.0)	29 (10.8)	76 (28.3)	12.59

Question #9—trust label information						
1. Certainly yes	18 (20.9)	8 (22.9)	12 (27.9)	9 (31.0)	9 (11.8)	
2. Yes	56 (65.1)	14 (42.9)	20 (46.5)	11 (37.9)	46 (60.5)	
3. Certainly no	0 (0)	3 (8.6)	1 (2.3)	0 (0)	2 (2.6)	
4. I am not sure	12 (14.0)	9 (25.7)	10 (23.3)	9 (31.0)	19 (25.0)	
	86 (32.0)	35 (13.0)	43 (16.0)	29 (10.8)	76 (28.3)	**23.67**⁣^∗^

Question #10—recycling & pollution						
1. Certainly yes	40 (46.5)	17 (48.6)	23 (53.3)	16 (55.2)	35 (46.1)	
2. It makes a positive input	44 (51.2)	15 (42.9)	17 (39.5)	13 (44.8)	39 (51.3)	
3. Certainly not	0 (0)	2 (5.7)	1 (2.3)	0 (0)	1 (1.3)	
4. I do not know	2 (2.3)	1 (2.9)	2 (4.7)	0 (0)	1 (1.3)	
	86 (32.0)	35 (13.0)	43 (16.0)	29 (10.8)	76 (28.3)	10.16

*Note*: The star associated with each bold entry corresponds with the statistical significance at the bottom of the table.

⁣^∗^*p* < 0.05, ⁣^∗∗^*p* < 0.01, and ⁣^∗∗∗^*p* < 0.001 indicate statistical significance.

**Table 7:** Logistic regression analysis for variables predicting decision to separate packaging garbage into paper, plastic, glass, and metals.

**Predictor**	**β**	**SE**	**Wald**	**E** **x** **p** (**β**)
Age				
10–19	0.508	0.659	0.632	1.662
20–29	0.400	0.423	0.894	1.492
40–49	0.750	0.507	2.188	2.118
50–59	0.538	0.883	0.371	1.712
60–69	0.017	1.859	0.000	1.017
Education				
Primary	**−1.320**⁣^∗∗^	1.395	0.895	0.267
Jr. high school	**-0.717**⁣^∗∗^	1.127	0.405	0.488
Undergraduate	0.183	0.732	0.063	1.201
Bachelor	0.104	0.765	0.019	1.110
Postgraduate	0.257	0.854	0.090	1.293
Gender				
Male	0.505	0.301	2.816	1.657
Income				
0.5–1 k	**−**0.539	0.509	1.121	0.584
1–1.5 k	0.179	0.569	0.099	1.196
2–2.5 k	**−**0.472	0.560	0.712	0.623
2.5 k above	**−**0.140	0.505	0.077	0.870
Environment conscious 1^a^	**−**0.722	1.132	0.407	0.486
Environment conscious 2^b^	**0.839**⁣^∗∗^	0.294	8.157	2.313
Environment conscious 3^c^	**0.631**⁣^∗^	0.321	3.868	1.879
Environment conscious 4^d^	0.878	0.750	1.373	2.407
Constant	-1.685	1.048	2.586	0.185
*R* ^2^ (Nagelkerke)	0.159			
*R* ^2^ (Cox & Snell)	0.117			
**−**2LL	320.071			
*χ* ^2^	33.076⁣^∗^			
*df*	19			

*Note*: Reference groups: 30–39 (age); highschool graduate (education); female (gender); 1.6–2 k (income). The star associated with each bold entry corresponds with the statistical significance at the bottom of the table.

^a^Environment conscious 1: influenced by environmentally friendly in deciding to purchase a new food product.

^b^Environment conscious 2: influenced by the environmental friendliness of package.

^c^Environment conscious 3: trust the truthfullness of labels stating that package is environmentally friendly.

^d^Environment conscious 4: a belief that package recycling reduces pollution.

⁣^∗^*p* < 0.05, ⁣^∗∗^*p* < 0.01, and ⁣^∗∗∗^*p* < 0.001 indicate statistical significance.

**Table 8:** Influence of age on purchasing decisions.

	**Age**						
	**<20**	**20–29**	**30–39**	**40–49**	**50–59**	**>60**	**χ** ^2^
Question # 1—packaging							
1. Packaging not important	1 (4.8)	12 (8.8)	7 (13.5)	5 (13.5)	4 (44.4)	2 (66.7)	
2. Packaging important	20 (95.2)	135 (91.2)	45 (86.5)	32 (86.5)	5 (55.6)	1 (33.3)	
	21 (7.8)	148 (54.8)	52 (19.3)	37 (13.7)	9 (3.3)	3 (1.1)	**20.35**⁣^∗∗^

Question # 2—market types							
1. Farmers' market	1 (4.8)	20 (13.5)	6 (11.5)	7 (18.9)	4 (44.4)	2 (66.7)	
2. Large grocery store	10 (47.6)	85 (57.4)	33 (63.5)	23 (62.2)	4 (44.4)	0 (0)	
3. Others	10 (47.6)	43 (29.1)	13 (25.0)	7 (18.9)	1 (11.1)	1 (33.3)	
	21 (7.8)	148 (54.8)	52 (19.3)	37 (13.7)	9 (3.3)	3 (1.1)	**21.18**⁣^∗^

Question #3—influence							
1. Advertisement	7 (33.3)	38 (25.9)	15 (28.8)	6 (16.2)	2 (22.2)	0 (0)	
2. Attractive packaging	3 (14.3)	23 (15.6)	2 (3.8)	2 (5.4)	0 (0)	1 (33.3)	
3. Label information	5 (23.8)	47 (43.0)	15 (28.8)	18 (48.6)	6 (66.7)	1 (33.3)	
4. Food safety	6 (28.6)	37 (25.2)	19 (36.5)	10 (27.0)	1 (11.1)	0 (0)	
5. Environmentally friendly	0 (0)	2 (1.4)	1 (1.9)	1 (2.7)	0 (0)	1 (33.3)	
	21 (7.8)	148 (54.8)	52 (19.3)	37 (13.7)	9 (3.3)	3 (1.1)	**38.07**⁣^∗∗^

Question #4—environment friendly							
1. No, is not important	0 (0)	8 (5.4)	3 (5.8)	5 (13.5)	2 (22.2)	1 (33.3)	
2. No price is important	5 (23.8)	53 (36.1)	15 (28.8)	12 (33.4)	4 (44.4)	1 (33.3)	
3. Yes, I prefer environ. friendly packaging	16 (76.2)	80 (54.4)	33 (63.5)	20 (54.1)	3 (33.3)	1 (33.3)	
4. Others	0 (0)	5 (3.4)	1 (1.9)	0 (0)	0 (0)	0 (0)	
	21 (7.8)	147 (54.8)	52 (19.3)	37 (13.7)	9 (3.3)	3 (1.1)	18.21

Question #5—packaging materials							
1. Glass	14 (66.7)	130 (87.8)	45 (86.5)	32 (86.5)	8 (88.9)	3 (100)	
2. Plastic	3 (14.3)	10 (6.8)	3 (5.8)	1 (2.7)	0 (0)	0 (0)	
3. Fabric	1 (4.8)	0 (0)	1 (1.9)	0 (0)	1 (11.1)	0 (0)	
4. Metal	0 (0)	0 (0)	1 (1.9)	0 (0)	0 (0)	0 (0)	
5. Others	3 (14.3)	8 (5.4)	2 (3.8)	4 (10.8)	0 (0)	0 (0)	
	21 (7.8)	147 (54.8)	52 (19.3)	37 (13.7)	9 (3.3)	3 (1.1)	26.74

Question #6—will paying more							
1. Will not pay more. Prefer low price pkg.	5 (23.8)	20 (13.6)	10 (19.2)	5 (13.9)	4 (44.4)	0 (0)	
2. Will choose environ pkg. if lower price	7 (33.3)	87 (59.2)	24 (46.2)	24 (66.7)	5 (55.6)	2 (66.7)	
3. Will to pay 10% more for environ pkg.	6 (28.6)	33 (22.4)	14 (26.9)	7 (19.4)	0 (0)	0 (0)	
4. Will to pay 20% more for environ pkg.	2 (9.5)	5 (3.4)	3 (5.8)	0 (0)	0 (0)	0 (0)	
5. Will to pay 30% more for environ pkg.	1 (4.8)	2 (1.4)	1 (1.9)	0 (0)	0 (0)	0 (0)	
	21 (7.8)	147 (54.8)	52 (19.3)	37 (13.7)	9 (3.3)	3 (1.1)	22.29

Question #7—recycling symbol							
1. Yes	18 (85.7)	131 (88.5)	40 (76.9)	27 (73.0)	5 (55.6)	1 (33.3)	
2. No	3 (14.3)	17 (11.5)	12 (23.1)	10 (27.0)	4 (44.4)	2 (66.7)	
	21 (7.8)	147 (54.8)	52 (19.3)	37 (13.7)	9 (3.3)	3 (1.1)	**16.63**⁣^∗∗^

Question #8—separate garbage							
1. Yes, all the time	3 (14.3)	16 (10.8)	5 (9.6)	7 (18.9)	0 (0)	1 (33.3)	
2. Sometimes	11 (52.4)	17 (52.0)	24 (46.2)	19 (51.4)	5 (55.6)	0 (0)	
3. No	7 (33.3)	55 (37.2)	23 (44.2)	11 (29.7)	4 (44.4)	2 (66.7)	
	21 (7.8)	147 (54.8)	52 (19.3)	37 (13.7)	9 (3.3)	3 (1.1)	8.20

Question #9—trust label information							
1. Certainly yes	5 (23.8)	28 (18.9)	12 (23.1)	10 (27.0)	1 (11.1)	0 (0)	
2. Yes	10 (47.6)	92 (62.2)	20 (38.5)	17 (45.9)	7 (77.8)	3 (100)	
3. Certainly no	0 (0)	2 (1.4)	4 (7.7)	0 (0)	0 (0)	0 (0)	
4. I am not sure	6 (28.6)	26 (17.6)	16 (30.8)	10 (27.0)	1 (11.1)	0 (0)	
	21 (7.8)	147 (54.8)	52 (19.3)	37 (13.7)	9 (3.3)	3 (1.1)	23.07

Question #10—recycling & pollution							
1. Certainly yes	13 (61.9)	73 (49.3)	21 (40.4)	21 (56.8)	3 (33.3)	0 (0)	
2. It makes a positive input	6 (28.9)	74 (50.0)	26 (50.0)	14 (37.8)	6 (66.7)	3 (100)	
3. Certainly not	0 (0)	1 (0.7)	3 (5.8)	0 (0)	0 (0)	0 (0)	
4. I do not know	2 (9.5)	0 (0)	2 (3.8)	2 (5.54)	0 (0)	0 (0)	
	21 (7.8)	147 (54.8)	52 (19.3)	37 (13.7)	9 (3.3)	3 (1.1)	**27.92**⁣^∗^

*Note*: The star associated with each bold entry corresponds with the statistical significance at the bottom of the table.

⁣^∗^*p* < 0.05, ⁣^∗∗^*p* < 0.01, and ⁣^∗∗∗^*p* < 0.001 indicate statistical significance.

## Data Availability

The data that support the findings of this study are available from the corresponding author upon reasonable request.

## References

[1] Jain S. K., Kaur G. (2004). Green Marketing: An Attitudinal and Behavioural Analysis of Indian Consumers. *Global Business Review*.

[2] Kumar R., Verma A., Shome A. (2021). Impacts of Plastic Pollution on Ecosystem Services, Sustainable Development Goals, and Need to Focus on Circular Economy and Policy Interventions. *Sustainability*.

[3] Thøgersen J. (2021). Consumer Behavior and Climate Change: Consumers Need Considerable Assistance. *Current Opinion in Behavioral Sciences*.

[4] White K., Hardisty D. J., Habib R. (2019). The Elusive Green Consumer. Harvard Business Review. https://hbr.org/2019/07/the-elusive-green-consumer.

[5] Carrión-Bósquez N., Veas-González I., Naranjo-Armijo F. (2024). Advertising and Eco-Labels as Influencers of Eco-Consumer Attitudes and Awareness-Case Study of Ecuador. *Foods*.

[6] Ramli N., Rashid N. A. (2009). Awareness of Eco-Label in Malaysia’s Green Marketing Initiative. *International Journal of Business and Management*.

[7] Ornella T.-O., Magdalena A., Fabiana S. R. P. (2024). Strengths and Weaknesses of Food Eco-Labeling: A Review. *Frontiers in Nutrition*.

[8] Barber N., Taylor D. C., Strick S. (2009). *Environmental Knowledge and Attitudes: Influencing the Purchase Decisions of Wine Consumers*.

[9] Zhang X., Dong F. (2020). Why Do Consumers Make Green Purchase Decisions? Insights From a Systematic Review. *International Journal of Environmental Research and Public Health*.

[10] Elmor L., Ramos G. A., Vieites Y., Andretti B., Andrade E. B. (2024). Environmental Sustainability Considerations (or Lack Thereof) in Consumer Decision Making. *International Journal of Research in Marketing*.

[11] Li J., Zhang J., Zhang D., Ji Q. (2019). Does Gender Inequality Affect Household Green Consumption Behaviour in China?. *Energy Policy*.

[12] Ziyue Z., Yuanchao G., Yang L., Linxiu Z., Yan S. (2021). Gender-Related Beliefs, Norms, and the Link With Green Consumption. *Frontiers in Psychology*.

[13] Tikka P. M., Kuitunen M. T., Tynys S. M. (2000). Effects of Educational Background on Students’ Attitudes, Activity Levels, and Knowledge Concerning the Environment. *Journal of Environmental Education*.

[14] Ng S., Bharti M., Faust N. T., Cheung F. M., Halpern D. F. (2020). The Impact of Gender and Culture in Consumer Behavior. *The Cambridge Handbook of the International Psychology of Women. Cambridge Handbooks in Psychology*.

[15] Schwartz J., Miller T. (1991). The Earth’s Best Friends. *American Demographics*.

[16] Newell S. J., Green C. L. (1997). Racial Differences in Consumer Environmental Concern. *Journal of Consumer Affairs*.

[17] Roberts J. A. (1996). Green Consumers in the 1990s: Profile and Implications for Advertising. *Journal of Business Research*.

[18] Qiao K., Dowell G. (2022). Environmental Concerns, Income Inequality, and Purchase of Environmentally-Friendly Products: A Longitudinal Study of US Counties (2010-2017). *Research Policy*.

[19] Yoo J., Park H., Kim W. (2018). Compromise Effect and Consideration Set Size in Consumer Decision-Making. *Applied Economics Letters*.

